# Minimal intervention in dentistry: which is the best approach for silorane composite restoration repairs?

**DOI:** 10.4317/jced.57640

**Published:** 2021-04-01

**Authors:** Rayssa-Ferreira Zanatta, Carlos-Rocha-Gomes Torres, Juliana-Boa-Sorte de Oliveira, Karen-Cristina-Kazue Yui, Amanda-Guedes-Nogueira Matuda, Stephanie-Ribeiro Lopes, Ana-Paula-Valente-Pinho Mafetano, Raquel-Pinto Campos, Alessandra-Bühler Borges, Cesar-Rogério Pucci

**Affiliations:** 1 DDS, Ms, PhD. Department of Restorative Dentistry, School of Dentistry, University of Taubaté, Taubaté, Brazil, and Department of Restorative Dentistry, Institute of Science and Technology, São Paulo State University-UNESP, São Paulo, Brazil; 2 DDS, PhD. Department of Restorative Dentistry, Institute of Science and Technology, São Paulo State University-UNESP, São Paulo, Brazil; 3 DDS, Ms. Department of Restorative Dentistry, Institute of Science and Technology, São Paulo State University-UNESP, São Paulo, Brazil; 4 DDS, Ms, PhD. Department of Restorative Dentistry, Institute of Science and Technology, São Paulo State University-UNESP, São Paulo, Brazil; 5 DDS, Ms, PhD Student. Department of Restorative Dentistry, Institute of Science and Technology, São Paulo State University-UNESP, São Paulo, Brazil; 6 DDS, Ms Student. Department of Restorative Dentistry, Institute of Science and Technology, São Paulo State University-UNESP, São Paulo, Brazil

## Abstract

**Background:**

This study aimed to evaluate surface treatments, adhesives and composites for repairing silorane based restorations.

**Material and Methods:**

One hundred and twenty truncated cones (2 mm smaller diameter and 4 mm larger diameter) made of silorane composite were divided in 12 groups according with the surface treatment (diamond bur and oxide aluminum abrasion), the adhesive (Adper Scothbond Multipurpose (3M ESPE), Ecusit (Voco) and Filtek P90 Adhesive (3M ESPE)). Each group was subdivided in two according with the composite used for repair (methacrylate and silorane). The repair was made with a second truncated cone build over the first one and bond strength assssed by tensile strength. Data were submitted to ANOVA 3-way and Tukey’s test (*p*<0.05).

**Results:**

There was difference only for the adhesives and the composites, with conventional adhesives (Adper Scothbond Mutipurpose) and methacrylate-based composites (Filtek Z350) presenting superior tensile strength compared to the silorane ones (P90 Adhesive system and composite).

**Conclusions:**

Therefore, it must be concluded that silorane composite can be repaired with methacrylate base composites and adhesives.

** Key words:**Silorane composites, composites, bond strength, minimal intervention.

## Introduction

The improvement of mechanical properties and the adhesion technology, summed with the aesthetic characteristic has made tooth-colored composites very popular in Restorative Dentistry (1). Mainly, this composites are made from methacrylate monomers which presents excellent physical and mechanical properties (2,3) and also an accepTable clinical performance, but is associate with disadvantages, such as shrinkage stresses (4), which leads to marginal staining, microleakage, secondary caries, cusp deformation and postoperative sensitivity (1,3). Silorane based composites were developed earlier in 2007 as an option for posterior teeth restoration. The composition of these composites is based from the reaction of oxirane and siloxane molecules (5), which results in a monomer with lower shrinkage during curing, and consequently lower stress generation (5-8), dynamic flexural strength, static elastic modulli, microhardness, wear resistance, biocompatibility and color stability (7,9-12).

Although silorane composites is not available in the market anymore, it was used for several years, so that many patients still present restorations made with this material in function nowadays. Despite the monomer system used in the composites, every restoration has a life time span and degradation is still expected after a time (11). Masticatory forces and the constant presence of acids and saliva leads to the composite wear and/or fracture formation that can interfere with its function and form.

In order to delay or avoid replacement cycles that can compromise the tooth survival, due further structure removal, the minimal intervention concept fold by the repair of partially defective restorations (13-15). Repair techniques depends of the material type and usually comprises surface cleaning and smoothening, followed by a surface pretreatment, such as mechanical sandblasting, roughening or etching and then the application of adhesives, silanes or composites (13,16). Mostly, repairs are made with composite in restorations presenting functional failures, such as partial loss of adjacent hard tissue, chipping of crows, marginal gaps, weather biological and aesthetic failures such as secondary caries and marginal discoloration usually demands change of the restoration (13,14).

As the clinical distinguish of silorane or methacrylate-based composites is not possible yet, is important to know the bonding behavior of them with different adhesives. Therefore, the aim of this study was to evaluate the tensile bond strength of conventional methacrylate composite to a silorane based composite after different surface treatments and using different adhesive systems. The null hypothesis is that conventional methacrylate composite present similar bonding to silorane based composite, independent of the adhesive system used and the surface treatment performed.

## Material and Methods

-Specimens preparation

**Figure 1:**
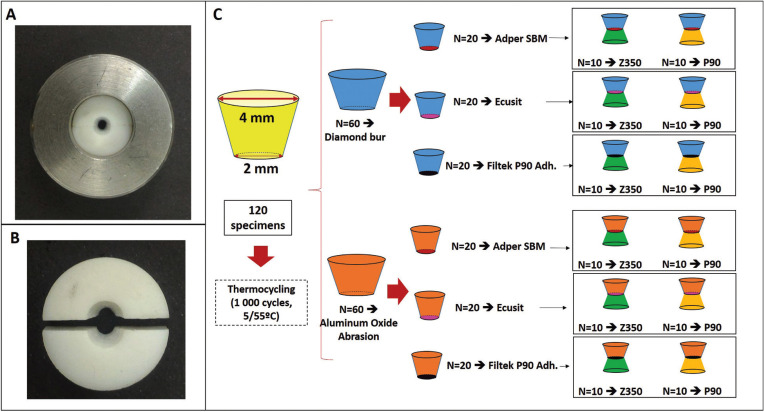
A) Teflon Split mould device with metallic ring used to build the composite specimens. B) Teflon device in higher augmentation. C) Dimensions of the conical shaped specimens and schematic drawing of groups division.

One hundred and twenty conical shaped specimens were made from silorane based composite (Filtek P90 – 3M ESPE, St Paul, MN, USA) using a two-piece Teflon device (Fig. 1A,B) as previously described (17). Each specimen presented 2 mm in the smaller diameter and 4 mm in the larger one (Fig. 1C) and were made in increments of 2 mm, which were inserted in the device and cured for 40 s (600 mW/cm2 XL 3000, 3M ESPE) each.

Specimens were aged in a thermocycling machine (10000 cycles, 5/55° C, with a dwell time of 25s and a transfer time of 5s) and then divided in two groups (n=60), according with the surface treatment performed: DB – smaller diameter surface abraded with a diamond bur (#1014 – KG Sorensen, Sao Paulo, Brazil); and AOA – smaller diameter surface sandblasted with 50μm aluminum oxide particles (Micro-etcher ERC, Danville Engineering, San Ramon, CA, USA) for 10 s, and distanced from 1 cm of the surface.

After the surface treatments, specimens from each group were subdivided in three other (n=20) subgroups according with the adhesive system used: conventional (Adper Scotchbond Multipurpose, 3M ESPE), indicated for composite repair (Ecusit, VOCO, Germany) and silorane based adhesive (Filtek P90 Adhesive, 3M ESPE). Each adhesive system was applied according manufactures instructions and are described in Table 1.

**Table 1:**
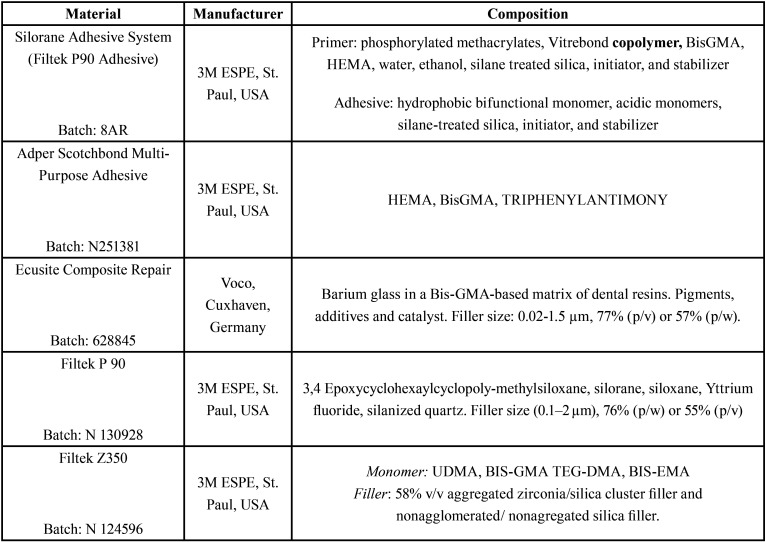
Composition of materials used in the study.

Finally, each subgroup was again subdivided (n=10) according with the composite type: conventional methacrylate composite (Filtek Z350, 3M ESPE) and silorane based composite (Filtek P90, 3M ESPE), as showed in Fig. 1C. Then, a second Teflon device was used to build another cone over the aged and treated one, using the respective composite of each group, leaving the final specimen with two truncated cones, adhered by the smaller diameter base, as previously described (17,18).

-Bond strength analysis

**Figure 2:**
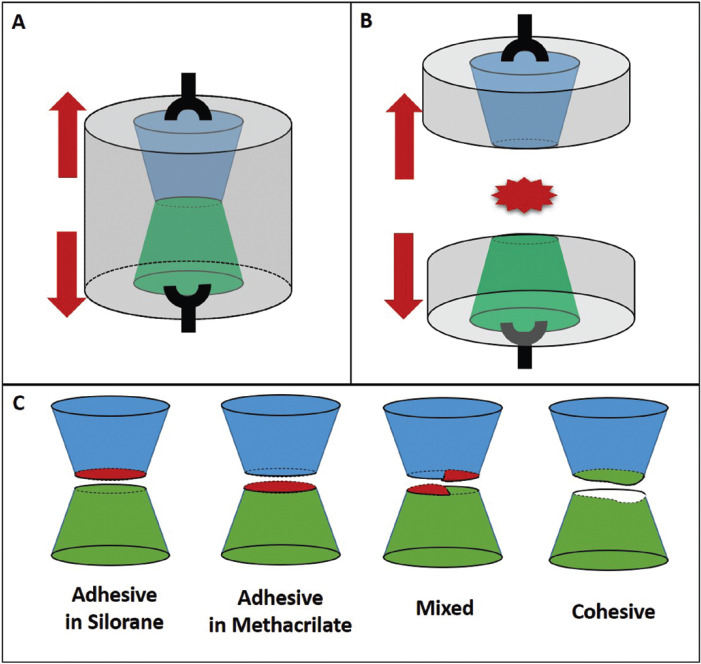
A) Schematic drawing of the conical specimens inside the metallic device. B) Separation of the split metallic device after the tensile test. C) Schematic drawing of the failures.

A metallic device was used to adapt the specimens and perform the tensile bond strength measurement (Fig. 2). This device guaranteed the correct alignment of the specimen in the universal testing machine (Emic DL-1000, São José dos Pinhais, PR, Brazil). The tensile test was performed with a 10 kg load cell at a crosshead speed of 1 mm/ min, according to the ISO 11405 Standard.

After bond strength measurement, specimens were analyzed according with the failure pattern at 50X magnification, under a stereomicroscope (Stemi 2000C, Zeiss, Carl Zeiss, Jena, Germany). The failure modes were classified as follows: adhesive in silorane, adhesive in methacrylate, mixed or cohesive (Fig. 2C).

-Statistical analysis

Data was checked for normality assumption (Kolmogorov Smirnov test) and analysis of variance in three levels (ANOVA 3-way) was performed using Statistica for Windows (version 7, Statsoft Inc, Tulsa, USA), considering surface treatment, adhesive system, and composite type as factors. Post hoc Tukey’s test was applied to check differences between groups (*p*<0.05).

## Results

The results of the ANOVA 3-way showed significant differences for the adhesive system factor (*p*<0.0001) and composite type (silorane or methacrylate) used (*p*<0.0001). No significant differences were found for the surface treatments (diamond bur or oxide aluminum abrasion) employed (*p*=0.319).

Regarding the adhesive system employed, Tukey test revealed that Ecusit presented the lower bond strength values, while Filtek P90 adhesive system present intermediary values and Adper Scotchbond Multipurpose presented the highest values. For the different composites tested, there was also significant differences (*p*<0.0001) between the groups, with the silorane one (Filtek P90) presenting lower values than the methacrylate (Filtek Z350). Table 2 shows the mean and standard deviation of all the groups tested. Regarding failure analysis, all failures were adhesives.

**Table 2:**
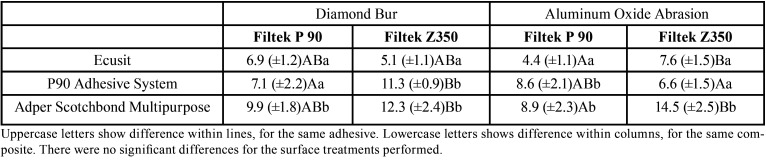
Mean and standard deviation for the bond strength (MPa) all the groups test.

## Discussion

The null hypothesis tested was denied for the adhesive system and the composite type, as the conventional methacrylate composite present similar bonding to silorane based composite, independent of the adhesive system used and the surface treatment performed. However, the hypothesis was accepted for the surface treatment, as no differences were found for bur abrasion or aluminum oxide abrasion.

Degradation of any tooth restorative material starts when it is put under function and it is expected over time (19). The main indications for composite repair is premature fractures, partial loss of restoration or adjacent tooth structure, need for re-contour, deficient contact point, infra-occlusion, inadequate anatomy, as well as chipping (13,20,21). Recently the restorative dentistry advocates for the minimal invasive philosophy, preferring the repair of partial function restoration than its complete change, aiming to avoid the weakening of the tooth structure by removal of the entire filling with burs (21). Studies have shown that repairing partially defective composites, instead of replacing them, are often performed(13) and reduces the risks and treatment costs (22), despite being also well accepted for patients and professionals (13).

Recently, clinical studies have shown that silorane composites do not present superior behavior than the methacrylate ones (23,24), and they were withdrawn from the market. Since it is clinically not possible to distinguish silorane composites from conventional methacrylate ones, as both are tooth-colored materials, restorations previously performed with these materials shall be able to be repaired with other materials. The results of this study show that this is possible, as similar bond strength values were obtained between the silorane and the conventional methacrylate composite (Table 2). Similar results were found by previous researchers (25,26), indicating that it is safe to make repair in this material using both methacrylate adhesives and composites. Even though some studies indicates that the conventional methacrylate composite presents a better bond strength reparability than silorane-based materials repaired with methacrylate resins (19,21). The lower repair potential of silorane resin found by these studies was attributed to the lower reactivity of silorane groups after polymerization. However, the present results show that the mechanical interlocking promoted by the surface treatments were able to improve the bonding area.

Regarding the surface treatment, there is no consensus about the best method for composite repair. Evidence suggests that sandblasting with aluminum oxide improves bond strength (25), but the results from this study showed no difference between the treatment with the aluminum blast or the diamond bur abrasion. This indicates that the mechanical interlocking is more critical than the mean used for making it in cases of composite repair. The similar results found in this study for diamond abrasion method and the sandblasting shows that clinicians can easily perform repairs in their practices, as diamond burs are a very common and low-cost instrument.

Concerning the bond strength method used, the truncated cones was proposed Barakat and Powers (27) and have been used ever since (17,18). Mostly, it aims to increase the probability of bond failure at the adhesive interface, minimizing failures caused by gripping the specimen. Also, it combines the advantages of micro-tensile tests by using a small bond surface area (diameter of 2 mm) and avoids the stresses formed during specimen preparation (18). The test yielded accepTable results, with all failures classified in adhesives, and no premature failures. Despite the favorable results, clinical studies should validate these findings.

## Conclusions

It must be concluded that for silorane composite fillings requiring repairs, conventional adhesives (Adper Scothbond Mutipurpose) and methacrylate based composites (Filtek Z350) presented superior tensile strength compared to the silorane ones (P90 Adhesive system and composite).
